# Editorial: Artificial Intelligence and the Future of Biosecurity

**DOI:** 10.3389/fmicb.2026.1940679

**Published:** 2026-08-24

**Authors:** Dov Greenbaum

**Affiliations:** 1 Department of Molecular Biophysics and Biochemistry, Yale University, New Haven, NY, United States; 2 Zvi Meitar Institute for Legal Implications of Emerging Technologies, Reichman University, Herzliya, Israel; 3 Harry Radzyner Law School, Reichman University, Herzliya, Israel; 4 Berkeley Law, University of California, Berkeley, Berkeley, CA, United States

**Keywords:** adaptive expertise, AI biosecurity, AI governance, biosecurity pedagogy, dual-use research, epistemic calibration, responsible research conduct, science education

## Teaching for a technology we cannot yet name: judgment, capability, and AI biosecurity

A biosecurity guideline written around a named list of dangerous sequences is, in a sense, obsolete the moment a generative model can design a functional equivalent that appears on no list. This is the familiar predicament of governing at the speed of the life sciences: our rules are almost always calibrated to yesterday's technology, and by the time a framework is drafted, debated, and adopted, the capability it was meant to address has often moved on. The contributions to this Research Topic wrestle with that lag directly, and many arrive at a shared and, we think, correct conclusion: that durable governance cannot be organized around specific tools. It must be organized around capabilities, leading indicators, and the reasoning we apply before a system is ever built.

We want to draw attention to a second domain in which the same lag operates, largely unexamined: education. If governance frameworks risk regulating the past, curricula risk teaching it. A scientific training program organized around the particular AI systems available today may produce graduates highly proficient in tools, interfaces, and workflows that will change substantially before their careers are fully underway.

Current tools should be taught. The risk is that curricula become technologically overfitted, treating fluency with the presently dominant instrument as though it were the underlying expertise. Because the consequential technologies of tomorrow, whether extensions of AI or something not yet anticipated, cannot be reliably predicted, curricular updating alone cannot solve the problem. The more durable response is to identify and cultivate the capacities that make a researcher effective across changing technological environments. We have argued elsewhere that emerging-technology education should therefore center on the ethical, legal, and social reasoning that outlasts any particular tool, rather than on fluency with the tools themselves (Greenbaum, 2022).

## From artifacts to capabilities, unevenly

The shift is real but uneven, and it is far from complete. Most of the operative regulatory infrastructure, from export controls to sequence-screening norms, still governs named artifacts: lists of agents, categories of pathogens, catalogs of controlled sequences. What the papers in this Research Topic document is not a field that has arrived at capability-based governance, but a discipline beginning to think past the artifact in front of it. Not every contribution rejects a specific technology, and several deliberately focus on one; but a recurring move runs through the Research Topic, away from governing named artifacts in isolation and toward governing capabilities, workflows, access points, and institutional responsibilities.

Luhachack et al. take the National Academies' notion of AI “capability uplift” and build it into a layered stack organized around observable indicators, new datasets, model performance, the erosion of build-and-test barriers, sketching how such indicators could support a monitoring dashboard rather than a list of named products. Holko reframes biosecurity as the governance of compositional workflows, arguing that risk no longer lives inside individual components but in how they combine, and that a system can remain compliant at every step while producing an outcome that is unsafe as a whole. Hanke et al. push the intervention point upstream, proposing a pre-development risk-benefit review for biological AI models: a reasoning discipline rather than a technology, applied before a model is ever trained. And Wang et al. foreground the hard asymmetry at the center of it all, an “evaluation bottleneck” in which novel biological sequences can be generated and optimized far faster than they can be assessed for danger.

The more operational contributions show how that orientation translates into concrete safeguards, an area in which the challenge of screening synthesized constructs has been recognized for some time (Puzis et al., 2020). Feldman et al. adapt know-your-customer practices from financial compliance into a three-tier access framework, institutional verification, output screening, and behavioral monitoring, that raises the cost of misuse without waiting for new legislation. Brackmann et al. survey layered technical safeguards spanning protein-sequence screening, DNA-order screening, and design-software controls, noting that AI-generated proteins can be functionally equivalent to known toxins while sharing little sequence similarity, which leaves homology-based screening blind by construction. Smith et al. propose Automated Laboratory Security Tiers for cloud and self-driving laboratories, keyed to latent capability: what a facility could produce if compromised, rather than what it currently handles. Sawaya et al. offer a genomic-data obfuscation method as a potential middle path, one intended to preserve population-level research utility while impeding full reconstruction of dangerous sequences. Aveggio et al. reject the innovation-vs.-security binary, illustrating through three project profiles where investment could produce mutual benefit. And Siddiqui and Pannu review a defensive technology, glycol-vapor air disinfection, and identify future opportunities for sensor-informed modeling and AI-enabled optimization of its deployment, a reminder of what all of this is ultimately for.

The practical responses vary widely, from upstream review and access controls to sequence obfuscation and defensive environmental technology. But the common demand is for governance that can adapt as the underlying instruments change. Capability-based governance is a demand for human judgment under uncertainty, so a governance architecture built on that principle can be only as strong as an education system that produces people able to exercise such judgment. That demand has a neglected educational counterpart.

## What the evaluation bottleneck teaches us about teaching

The articles do not themselves offer a theory of education. Read together, however, they expose an educational dependency that biosecurity governance has not adequately addressed.

There are, famously, known knowns, known unknowns, and unknown unknowns, and it is the last category that tends to cause the most trouble (U.S. Department of Defense, 2002). The evaluation bottleneck that Wang et al. foreground is sharper than the familiar worry about an AI producing a plausible but mistaken answer, and it lives partly in that last category. The biological meaning of a generated sequence may remain uncertain until it is physically synthesized and expressed, so the act of resolving that uncertainty can itself create danger. Generation has outrun validation, and validation is slow as well as potentially hazardous.

A second problem is more familiar. Language models fail persuasively, returning answers that look authoritative to anyone without the background to catch the error. A researcher now faces uncertainty from two directions at once: outputs whose real-world function cannot be cheaply or safely confirmed, and outputs whose confident presentation invites acceptance without confirmation. The human contribution sits at their intersection, the judgment to recognize what is not yet known, to distinguish a known unknown from an unknown unknown, to demand appropriate validation rather than trust an authoritative-sounding result, and to resist unwarranted confidence in the model or in oneself. No tool confers this. It is a habit of mind, and a tool-specific curriculum does not build it.

The choice is not between teaching current tools and teaching durable judgment, but between teaching tools as ends in themselves and teaching them as the context in which transferable judgment develops. A researcher learns dual-use reasoning at a real bench, epistemic humility by being burned by a real model's confident error, systems thinking by tracing a real workflow. The academic model does not need to be discarded; it needs to shift its center of gravity from the instrument to the reasoning.

The organizing distinction is between routine and adaptive expertise. Routine expertise is growing faster and more fluent at today's tasks with today's instruments; adaptive expertise is the capacity to invent a new approach when the current one fails. The first depreciates in a field defined by instrumental obsolescence; the second survives it. Adaptive expertise rests on metacognitive flexibility and a first-principles grasp of one's field, the logic of verification, the structure of valid evidence, the anatomy of error, and it is not a fixed trait one either has or lacks. It can be deliberately cultivated, which is precisely why it belongs in a curriculum.

We can now name three primary competencies. The first is *dual-use reasoning as a reflex*, the instinct Hanke et al. would formalize into upstream review, applied at the bench as the habit of asking how a result could be misused at design time rather than at review time. The second is *epistemic calibration*, a working sense, in the spirit of Brackmann et al. screening critique, of where models fail, of the fact that they fail convincingly rather than obviously, and of when a result must be validated rather than believed. The third is *compositional, systems-level thinking*, Holko's insight turned into a personal skill, the capacity to reason about how tools, data, and workflows interact, since that is where both value and risk now originate. Two further competencies are what allow the first three to travel between technologies: *cross-domain literacy*, enough fluency across biology, computation, law, and ethics to work in teams that span them, a gap that has long impeded the very collaborations biosecurity now requires (Klein et al., 2017), and *adaptive expertise* itself, the capacity to apply first principles to whatever new instrument emerges. Future-proofing a curriculum by chasing the newest tool is, in this light, a category error; it assumes tomorrow's instruments will be linear extensions of today's, when scientific history suggests the opposite.

None of these is new as an educational aspiration. What is new is the cost of neglecting them.

## Why the stakes are higher here

In most fields, a graduate trained on outdated tools is merely inefficient; they catch up. Biosecurity offers a narrower margin. The same democratization this Research Topic examines, the cloud and self-driving laboratories Smith et al. analyze, the open pathogen sequence Sawaya et al. work to protect, the generative design tools Wang et al. and Brackmann et al. describe, means that a researcher who lacks dual-use reasoning is no longer just behind the curve. They may become a liability, capable, with today's automated infrastructure, of harm an equivalently unreflective researcher a decade ago could not have caused. Lowered barriers cut both ways: they widen access to good work, and they raise the price of poorly trained judgment.

This is why the pedagogical question cannot be fully separated from the governance one. Many of the frameworks in this Research Topic ultimately depend on a person who can catch a plausible but wrong output in a dual-use context: the reviewers Hanke et al. would convene, weighing a risk-benefit case; the institutional gatekeepers Feldman et al. rely on; the laboratory staff who must recognize an anomalous order; the designers who pause at a dual-use inflection point. That is demanding work, a sustained exercise of high-stakes judgment over exactly the kind of confident, persuasive output a model produces. And here the two lags meet. If the next generation is trained by outsourcing understanding to the model, retaining the answer while losing the capacity to interrogate it, then the human layer on which those safeguards rest is quietly hollowed out. A failure of pedagogy does not merely produce weaker researchers; it erodes the judgment the governance architecture was built to depend on.

## A skill set agnostic to the technology

The principle is not confined to the laboratory. Law, policy, medicine, and other fields being reshaped by AI face the same structural problem: educate for the technology of the moment and you produce practitioners fitted to a world that is already dissolving. Each will have to ask, in its own terms, what the technology-agnostic core of its expertise actually is.

For AI-enabled biology, this Research Topic has done the harder half of that work already, showing what capability-based, anticipatory, tool-independent governance can look like. The task it sets for the field next is to build the matching pedagogy: to train students, scientists, and reviewers not in the instruments of today but in the durable capacities that will let them govern, and use, the instruments we cannot yet name. We do not fail our researchers by teaching them too little about any particular tool. We fail them if we do not teach them how to think when the tool is confidently wrong, and no one in the room knows enough to notice. That, not fluency with any model, is the capability on which safe biology now depends.
